# Comparison between Novel Anatomical Locking Guide Plate and Conventional Locking Plate for Acetabular Fractures: A Finite Element Analysis

**DOI:** 10.3390/life13112108

**Published:** 2023-10-24

**Authors:** Xiao Liu, Jianpeng Gao, Xiaoyong Wu, Junhao Deng, Zijian Li, Ran Li, Licheng Zhang, Jianheng Liu, Ming Li

**Affiliations:** 1 Department of Orthopaedics, The First Medical Center of the Chinese PLA General Hospital, No. 28 Fuxin Road, Beijing 100853, China; liuxiao23_314@163.com (X.L.); bonegaojp@163.com (J.G.); wxy1999_2008@hotmail.com (X.W.); deng_junh@163.com (J.D.); lizijian9624@126.com (Z.L.); liran21153@163.com (R.L.); zhanglcheng218@126.com (L.Z.); 2 National Clinical Research Center for Orthopedics, Sports Medicine & Rehabilitation, No. 28 Fuxin Road, Beijing 100853, China

**Keywords:** acetabulum, internal fixators, fractures, finite element analysis

## Abstract

The treatment of complex acetabular fractures remains a complicated clinical challenge. Our self-designed novel anatomical locking guide plate (NALGP) has previously shown promising potential in T-shaped acetabular fractures (TAF), but a direct comparison with conventional fixations is yet to be made. The TAF model was established based on a volunteer’s computer tomography data and then fixed with double column locking plates (DLP), a posterior column locking plate with anterior column screws (LPACS), and our NALGP. Forces of 200 N, 400 N, and 600 N were then loaded on the model vertically downward, respectively. The stress distribution and peaks and maximum displacements at three sites were assessed. We found that the stress area of all three plates was mainly concentrated around the fracture line, while only the matching screws of the NALGP showed no obvious stress concentration points. In addition, the NALGP and DLP showed significantly less fracture fragment displacement than the LPACS at the three main fracture sites. The NALGP was found to have less displacement than DLP at the posterior column and ischiopubic branch sites, especially under the higher loading forces of 400 N and 600 N. The fixation stability of the NALGP for TAF was similar to that of DLP but better than that of LPACS. Moreover, the NALGP and its matching screws have a more reasonable stress distribution under different loads of force and the same strength as the LPACS.

## 1. Introduction

Recently, with the development of a mechanized society and an increasingly aging population, the incidence of acetabular fracture has risen [1,2]. To address complex acetabular fractures, such as those that are T-shaped, the most effective approach is to employ rigid internal fixation utilizing a range of internal fixation devices, which is widely accepted as the definitive treatment [3,4,5,6]. However, achieving satisfactory anatomical reduction and fixation is challenging considering the complexity of the anatomy of the pelvis [7,8].

The development of novel internal fixation devices for acetabular fractures has been the focus of in-depth and ongoing study [9], including locking plates, which have become increasingly popular in recent years [10,11]. In particular, the locking steel plate achieves a secure connection to the screw via its locking screw hole. This offers angular stability, screw extraction resistance, and single cortical fixation, resulting in a more stable and reliable bone fixation in orthopedic surgeries [12]. At present, the customary surgical intervention for intricate acetabular fractures that implicate both the anterior and posterior columns, such as T-shaped acetabular fractures (TAF), is to secure the double columns with steel plates [13,14]. These classic double column locking plates (DLP) can provide sufficient biomechanical stability. However, a combined anterior and posterior approach is required [15], which may result in extensive secondary trauma and more postoperative complications [16,17,18,19]. The incidence of heterotopic ossification after acetabular surgery is as high as 25.6% [20,21], which may be inextricably linked to the complexity of surgical incision. Therefore, an increasing number of surgeons have begun to use a single-incision surgical approach that can fix the plate to one column through a unilateral approach and fix the opposite column using lag screws [22,23,24,25,26]. For example, a posterior column locking plate with anterior column screws (LPACS) showed reduced trauma using the single-incision approach [27]. Nonetheless, the biomechanical strength and stability of this internal fixation mode may be less than DLP fixation [28]. Furthermore, there are fewer anatomical landmarks for reference when inserting the anterior column lag screw, increasing the difficulty of surgery.

Additionally, the morphology of the acetabulum surface is very complicated due to the particular structure of the pelvic bone [29]. Therefore, the plates of the two fixation methods mentioned above cannot be completely attached to the acetabulum. Since the direction of the locking plate screw is fixed, excessive pre-bending and shaping of the steel plate can cause the screw to deviate from the expected direction, which would increase the possibility of the screw penetrating into the pelvic cavity and damaging important organs. Moreover, surgeons may be forced to abandon the locking screw due to thread deformation, which may affect the strength of the fixation and even lead to the fracture shifting again [30].

Previously, we established a systematic database of Chinese acetabular morphology based on cloud data collected from 171 computed tomography (CT) models [31,32]. This database was employed to standardize measurements and calculations for various acetabular, ultimately resulting in the determination of the average morphology of the posterior column surface of the acetabulum [number ZL200910087252.1, CN PAT]. Following this, our team designed and manufactured a novel anatomical locking guide plate (NALGP) with screw-hole threads for the treatment of complex acetabular fractures involving both columns [number ZL201620858267.9, CN PAT]. Specifically, using a minimally invasive single posterior approach, the NALGP can be strictly attached to the posterior column surface without pre-bending and shaping. When the NALGP is attached, various screws can be inserted into the screw holes at pre-determined angles, greatly reducing the potential for damage to the joints and proximal soft tissues. In accordance with the findings of Gusic et al. [33], it facilitates stable screw fixations in the ideal acetabular sites and can provide strong internal fixation through a single posterior approach. Furthermore, the NALGP could provide adequate fixation of both the anterior and posterior columns simultaneously through the use of two types of screws (anterior column screws and magic screws). An inverted Y-shaped structure would then be formed by the NALGP and two screws to maximize the reconstruction of the normal acetabular anatomical structure [34,35]. 

No direct comparison between our novel plate and traditional internal fixations in TAF has been conducted to date. Moreover, research models for the evaluation of biomechanical stability have mostly focused on several easily modeled fracture types, whereas research on complex acetabular fractures involving two columns is scarce. Consequently, here, we utilized the finite element method to establish a representative TAF model. Then we determined which treatment method was more conducive to anatomical reduction and strong fixation after TAF among DLP, LPACS, and NALGP.

## 2. Methods

### 2.1. Three-Dimensional Modeling of the TAF

**Figure 1:**
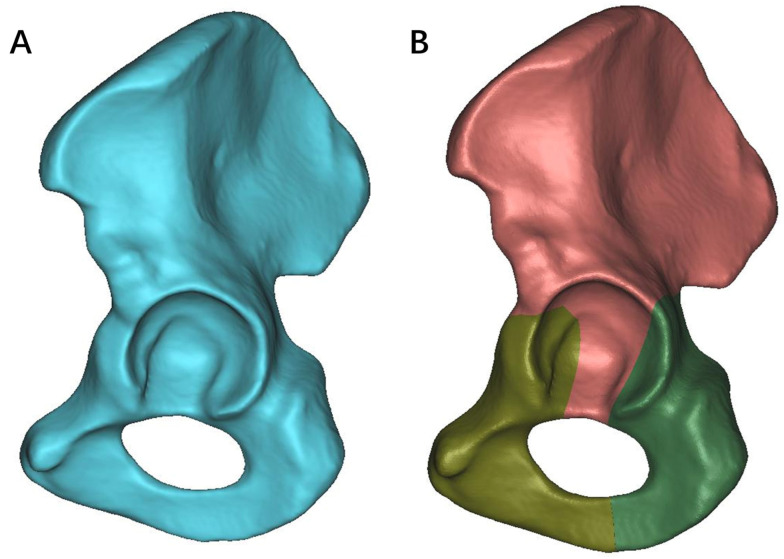
Three-dimensional models of both normal acetabulum and T-shaped acetabular fractures (TAF): (**A**) The normal acetabulum model (half pelvis) is derived from volunteer imaging data. (**B**) The TAF model was constructed according to the Judet and Letournel classification standards. Fracture lines and fragments are differentiated by different colors.

The DICOM data from a 48-year-old male volunteer’s CT were imported into Mimics software (version 16.0, Materialise Company, Leuven, Belgium); coronal, sagittal, and transverse planes were defined. The software’s dynamic area growth method was used to establish a three-dimensional model of the normal acetabulum (half pelvis) and TAF [36] (Figure 1A). This accurate geometric model was built based on the bone contour in Mimics as previously stated [37,38]. 

The TAF model was established according to the Judet and Letournel classification standards [39]. In brief, the first fracture line started below the anterior inferior iliac spine, involving the anterior one-third articular surface of the acetabular fossa, and extended downward to the junction of the pubic ischia. The second fracture line started below the ischial notch, involving the posterior one-third of the articular surface of the acetabular fossa, and extended downward to the junction of the pubis and ischia [14,40] (Figure 1B). 

### 2.2. Geometric Modeling of Different Internal Fixations for TAF

**Figure 2:**
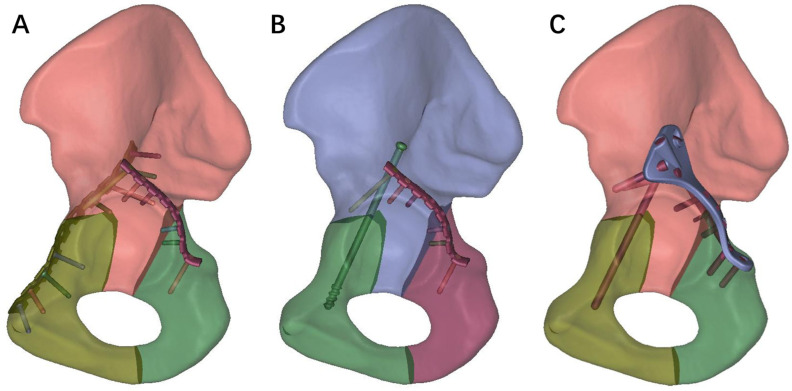
The construction of three distinct internal fixation models for TAF. (**A**) Double column locking plates (DLP), (**B**) Posterior column locking plate with anterior column screws (LPACS), (**C**) Novel anatomical locking guide plate (NALGP).

Three internal fixation models, namely DLP, LPACS, and NALGP, were created using the Unigraphis software (Unigraphis Solutions of EDS, Torrance, CA, USA) and are depicted in Figure 2. The model file assemblies were imported into Hypermesh 10.0 (Altair, Troy, MI, USA) to perform mesh division and volume network transformation. Grid convergence analysis was conducted for various sizes, and the resulting nodes and elements for the different internal fixation methods are presented in Table 1.

### 2.3. Definition of the Boundary Conditions, Material Properties, and the Loading Modes

The boundary conditions, material properties, and loading force were defined using Abaqus 6.14 software (Dassault Systems, Velizy-Villacoublay, France). Binding was applied to the contact surfaces between the bone and internal fixation or between devices, while the contact surface between fracture fragments was defined as friction with a friction coefficient of 0.2 [41]. Furthermore, the material properties were established by consulting the pertinent literature [42]. It was postulated that cortical bones, cancellous bones, and internal fixations were all characterized as continuous and isotropic linear elastic materials. The specific material properties can be found in Table 2.

To facilitate computations, we implemented a rigid fixation of the pubic symphysis and sacroiliac joint. The model’s boundary condition was established by restricting the ipsilateral ischial tuberosity and constraining its six degrees of freedom. Patients with TAF are usually required to perform early partial or complete weight-bearing, thus each assembly model was subjected to vertical loads of 200 N, 400 N, and 600 N, representing the forces experienced during partial weight-bearing [43,44], sitting [28], and standing postures [45] following surgical intervention. The loading force was directed 45° upward in the coronal plane and 25° backward in the sagittal plane.

### 2.4. Evaluation Index of Three Internal Fixation Methods

The stress distribution, stress peak of the plates and screws, and the displacement of the fracture ends were assessed under various vertical loads. The stress distribution and peak values serve as comprehensive indicators of the strength of these instruments, aiding in the identification of appropriate materials. Furthermore, the displacement of fracture fragments provides a reliable measure of the stability of internal fixation in TAF [41].

## 3. Results

### 3.1. Stress Distribution and Stress Peak

**Figure 3:**
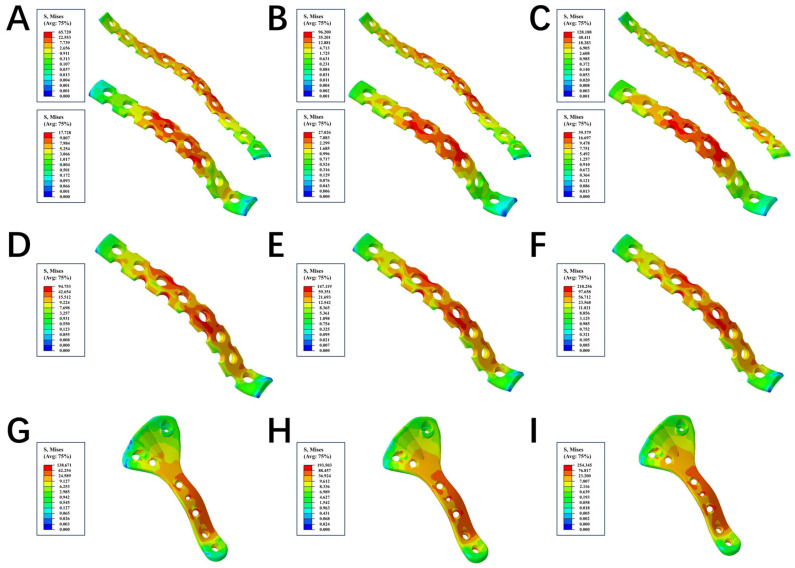
The stress nephogram of plate among the three groups. (**A**–**C**) represent the DLP group under the loading force of 200 N, 400 N, and 600 N, respectively. The top of (**A**–**C**) showed the anterior column plate, while the bottom showed the posterior column plate. (**D**–**F**) represent the LPACS group under the loading force of 200 N, 400 N, and 600 N, respectively. (**G**–**I**) represent the NALGP group under the loading force of 200 N, 400 N, and 600 N, respectively. The red regions in the plate endured the maximum force, while the blue area underwent the minimum force.

**Figure 4:**
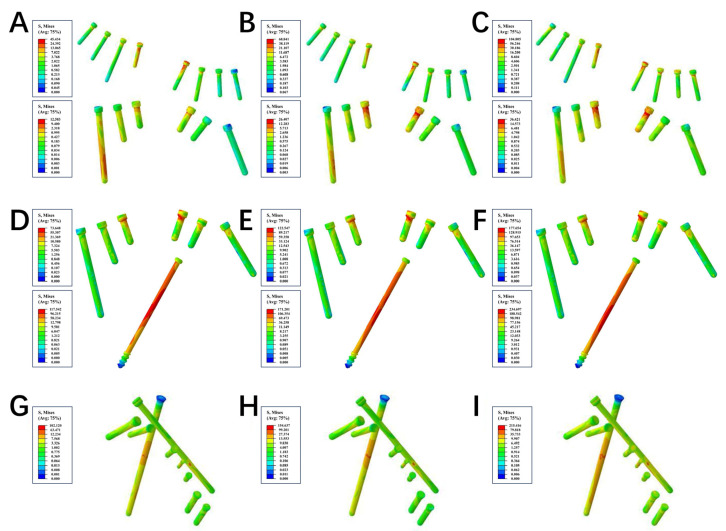
The stress nephogram of screws among the three groups. (**A**–**C**) represent the DLP group under the loading force of 200 N, 400 N, and 600 N, respectively. The top of (**A**–**C**) showed the anterior column screws, while the bottom showed the posterior column screws. (**D**–**F**) represent the LPACS group under the loading force of 200 N, 400 N, and 600 N, respectively. (**G**–**I**) represent the NALGP group under the loading force of 200 N, 400 N, and 600 N, respectively. The red regions in the screw endured the maximum force, while the blue area underwent the minimum force.

Initially, we conducted an assessment on the strength of the three internal fixations, and we observed significant differences in the stress distributions of both plates and screws. Under the loading forces of 200 N, 400 N, and 600 N, the von Mises stress peaks of the plate in the LPACS were 94.753 MPa, 147.119 MPa, and 210.256 MPa, respectively; the von Mises stress peaks of the anterior and posterior plates in the DLP were 65.72 and 17.728 MPa, 96.2 and 27.026 MPa, and 128.188 and 39.379 MPa, respectively; and the stress peaks of the novel plate in the NALGP were 138.671 MPa, 193.503 MPa, and 254.345 MPa, respectively (Table 3 and Figure 3). For the screws under the same loading forces (200 N, 400 N, and 600 N), the stress peaks of the anterior and posterior screws in the LPACS were 117.542 and 73.648 MPa, 171.201 and 122.547 MPa, and 234.697 and 177.654 MPa, respectively; in the DLP were 45.434 and 12.583 MPa, 68.841 and 26.407 MPa, and 104.805 and 36.421 MPa, respectively; and in the NALGP were 102.12 MPa, 154.637 MPa, and 215.416 MPa, respectively (Table 4 and Figure 4).

### 3.2. Displacement

Next, we evaluated the maximum displacement of the three main fracture sites, including the anterior column fracture (ACF) line, posterior column fracture (PCF) line, and ischia pubic branch fracture (IPBF) line, under the same loads. 

**Figure 5:**
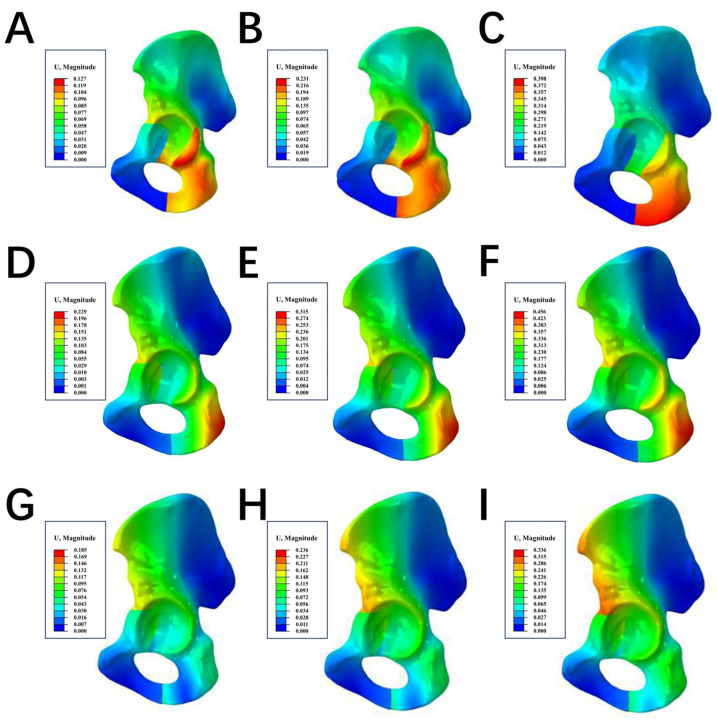
The maximum displacement of fractures at three sites. (**A**–**C**) represent the DLP group under the loading force of 200 N, 400 N, and 600 N, respectively. (**D**–**F**) represent the LPACS group under the loading force of 200 N, 400 N, and 600 N, respectively. (**G**–**I**) represent the NALGP group under the loading force of 200 N, 400 N, and 600 N, respectively. The red regions in the hip endured the maximum displacement, while the blue area underwent the minimum displacement.

At the site of the ACF line under loads of 200 N, 400 N, and 600 N, the maximum displacements in the LPACS were 0.164 mm, 0.255 mm, and 0.367 mm, respectively; in the DLP were 0.09 mm, 0.188 mm, and 0.274 mm, respectively; and in the NALGP were 0.145 mm, 0.215 mm, and 0.326 mm, respectively. At the PCF site under the same loading forces (200 N, 400 N, and 600 N), the maximum displacements in the LPACS were 0.158 mm, 0.249 mm, and 0.352 mm, respectively; in the DLP were 0.126 mm, 0.227 mm, and 0.306 mm, respectively; and in the NALGP were 0.113 mm, 0.157 mm, and 0.216 mm, respectively. Similarly, the maximum displacements under loads at the IPBF in the LPACS were 0.13 mm, 0.225 mm, and 0.341 mm, respectively; in the DLP were 0.117 mm, 0.223 mm, and 0.338 mm, respectively; and in the NALGP were 0.124 mm, 0.152 mm, and 0.195 mm, respectively (Table 5 and Figure 5). 

## 4. Discussion

The main features of TAF are the fracture line’s involvement of the anterior and posterior columns of the acetabulum, that the fractured component loses connection with the axial bone, and that both columns are separated from each other [2,43]. Ye et al. [14] overlaid and plotted the fracture lines of 56 T-shaped fractures on a template, then generated frequency heat maps based on the density of the fracture line distribution. They concluded that T-shaped fracture lines resembled a “Y” shape rather than a “T” shape. Based on the distribution area of the high frequency of the fracture line as depicted on the heat map, we established a representative TAF model [14], which conforms to the classification standards of Judet and Letournel [39]. It was formed by two independent fracture lines of disjoint anterior and posterior columns formed in the acetabular fossa. It was a complete low-position intra-articular fracture involving double columns possessing extremely unstable biomechanics, which is consistent with one of the typical fracture lines of the TAF described by Becker et al. [43].

Subsequently, using the finite element analysis method [46], we compared the differences in stress distribution and stress peak between the NALGP, the traditional DLP, and the recently widely popular LPACS. Subsequently, we recorded the stress distributions along with the stress peaks of the steel plates and screws for analysis. The stress distribution can be used to indicate the ability of plates or screws to resist elastic deformation when subjected to force, as stress concentration may cause the plate or screw to deform or even fracture [47]. Under the different loading forces, it can be observed from the stress nephogram that the stress area of all three plates is mainly concentrated around the fracture line, while only the screws of the NALGP have no obvious stress concentration points. This means that the stress distribution of the NALGP is more reasonable and uniform than that of the other two internal fixation plates and screws. Furthermore, the anterior screw in the LPACS bears the greatest stress regardless of load, followed by the matching screws in the NALGP. Under the same load, the NALGP bears the greatest stress, followed by the LPACS, indicating that the NALGP system can provide sufficient strength. 

We compared the maximum displacement of the three main fracture sites under different loads to assess the stability of different internal fixations. It is well known that displacement is one of the attributes that reflects the stability of implants. As expected, the DLP had the smallest anterior column displacement, owing to the greater fixation strength when the plate was fixed directly to the front column [48]. Regarding the two indirect fixation systems, the NALGP maintained less displacement than the LPACS, suggesting that it is a more stable indirect fixation method. Notably, in the posterior column, the displacement of the NALGP was significantly smaller than that of the others, indicating that the NALGP has the best fixation stability for the posterior column. This may be related to the posterior magic screw of the NALGP. The ischiopubic displacement was dependent on load. Under a load of 200 N, the DLP exhibited the smallest displacement. However, with an increase in loading force, the NALGP showed the least displacement. This illustrates that as the loading force increased, the stability advantages of the new steel plate became more obvious. It should be emphasized that the fixation of the ischiopubic branch was achieved indirectly as a result of fixing both columns. Therefore, the displacement of the ischiopubic branch directly reflects the stability of the entire internal fixation device. In other words, the integral inverted “Y”-shaped fixing structure formed by the anterior column lag screw and the posterior magic screw of the NALGP has the strongest fixing effect. Mechanobiology is crucial for fracture healing. Plates typically have a low bending stiffness, leading to the axial compression of interfragmentary movement (IFM), which may impact fracture healing [49]. Our research showed that all three groups of internal fixation devices had a fracture gap displacement of less than 1 mm at varying loads, indicating no detrimental influence on fracture healing in mechanobiology.

In recent years, locking plates have emerged as a novel method for the treatment of acetabular fractures owing to their superior angular stability and monocortical screw fixation. However, because the direction of the screw is fixed, excessive pre-bending and shaping of the steel plate will cause the screw to deviate from the expected direction, which increases the possibility that the screw penetrates the pelvic cavity and damages important organs. Moreover, surgeons may be forced to abandon the locking screw owing to thread deformation, which may affect the strength of the fixation and even lead to fracture shifting again [30].

To overcome these weaknesses, our team designed and manufactured an NALGP. Specifically, its shape was designed by extracting a large amount of Chinese acetabular posterior column surface morphology data, so it could be strictly attached to the surface of the fracture site. There is no need to pre-bend and shape the plate during the operation, guaranteeing the strength of the steel plate. The centerline angle of the screw hole on the steel plate was obtained based on an analysis of a large amount of sample data. The preset nail placement direction can accurately avoid important arteries, nerve plexuses, and other important tissues; thus, the nail can be directly placed safely and efficiently. In theory, the application of the NALGP could shorten operation time and reduce soft tissue damage, perioperative bleeding, and incidence of surgical complications. Magic screws, which are renowned for their challenging insertion, are indispensable screws for repairing the posterior column and quadrilateral plate. The NALGP’s preset magic screw nail is designed to enable the precise placement of the entry point on the gluteus medius eminence, located above the acetabulum and directed toward the ischial spine, which allows for accurate entry of the magic screw into the secure channel of the posterior column by the guiding pins. In addition, the magic screws and anterior column lag screws form an inverted Y-shaped structure that matches the normal mechanics of both acetabular columns.

This study has some limitations. First, the modeling of TAF is based on low-position both-column fractures, which cannot represent all T-sharp fracture models. In a follow-up study, more common fracture models will be adopted for further analysis. Second, as a controversial clinical conundrum, TAF also has other types of fixation methods. Further comparisons should be made in future studies. Third, the finite element model was simplified by disregarding the influence of positional changes and peri-acetabular soft tissues on the fixation pattern. Consequently, the fixation support of the model does not accurately represent the physiological condition. Fourth, the NALGP was designed by extracting a large amount of dataset that pertains to the Chinese acetabular posterior column surface morphology. Consequently, it is crucial to establish a more expansive database encompassing diverse ethnic groups, which would improve the design of the NALGP.

## 5. Conclusions

The fixation stability of the NALGP for TAF was similar to that of the DLP, and better than that of the LPACS. Moreover, the NALGP and its matching screws have a more reasonable stress distribution under different loads and the same strength as the LPACS. Of note, the NALGP can also complete both-column fixation through a single posterior approach similar to the LPACS, so its surgical trauma is theoretically less than DLP fixation. Overall, the NALGP provides a new and effective internal fixation method for the treatment of TAF, especially with relatively stable anterior column fractures. 

## Figures and Tables

**Table 1:** The statistics of nodes and elements with or without instruments fixation.

Model	Acetabular		Internal Fixations	
	Nodes	Elements	Nodes	Elements
DLP	188916	1006841	203938	122873
LPACS	187144	1000072	273578	178458
NALGP	188507	1001865	314224	204542

DLP: Double column locking plates; LPACS: Posterior column locking plate with anterior column screws; NALGP: Novel anatomical locking guide plate.

**Table 2:** Bone and internal fixation material properties.

Material Type	Elastic Modulus (MPa)	Poisson’s Ratio
Cortical bone	12,400	0.3
Cancellous bone	77	0.3
Instrument	110,000	0.3

**Table 3:** The von Mises stress peak of plate among the three groups.

		Stress (MPa)	
	200 N	400 N	600 N
DLP (anterior plate)	65.72	96.2	128.188
DLP (posterior plate)	17.728	27.026	39.379
LPACS (posterior plate)	94.753	147.119	210.256
NALGP (posterior plate)	138.671	193.503	254.345

**Table 4:** The von Mises stress peak of screws among the three groups.

		Stress (MPa)	
	200 N	400 N	600 N
DLP (anterior screw)	45.434	68.841	104.805
DLP (posterior screw)	12.583	26.407	36.421
LPACS (anterior screw)	117.542	171.201	234.697
LPACS (posterior screw)	73.648	122.547	177.654
NALGP (posterior screw)	102.12	154.637	215.416

**Table 5:** The maximum displacement of fracture at three sites among the three groups.

	The Maximum Displacement (mm)								
	ACF			PCF			IPBF		
	200 N	400 N	600 N	200 N	400 N	600 N	200 N	400 N	600 N
DLP	0.090	0.188	0.274	0.126	0.227	0.306	0.117	0.223	0.338
LPACS	0.164	0.255	0.367	0.158	0.249	0.352	0.130	0.225	0.341
NALGP	0.145	0.215	0.326	0.113	0.157	0.216	0.124	0.152	0.195

ACF: Anterior column fracture line; PCF: Posterior column fracture line; IPBF: Ischia pubic branch fracture line.

## Data Availability

The data that support the findings of this study are available from the corresponding author upon reasonable request.

## Abbreviations

TAF: T-shaped acetabular fractures; DLP: Double column locking plates; LPACS: Posterior column locking plate with anterior column screws; NALGP: Novel anatomical locking guide plate; ACF: Anterior column fracture; PCF: Posterior column fracture; IPBF: Ischia pubic branch fracture.
